# A Review of High-Throughput Optical Sensors for Food Detection Based on Machine Learning

**DOI:** 10.3390/foods15010133

**Published:** 2026-01-02

**Authors:** Yuzhen Wang, Yuchen Yang, Huilin Liu

**Affiliations:** 1 Key Laboratory of Digital-Intelligence and Dynamic Perception for Food Quality of China Light Industry, Beijing Technology and Business University, Beijing 100048, China; 20240900@btbu.edu.cn (Y.W.); yangyc202209@163.com (Y.Y.); 2 Beijing Laboratory for System Engineering of Carbon Neutrality, Beijing Municipal Education Commission, Beijing 100048, China

**Keywords:** digital and intelligent optical sensors, food inspection, machine learning, nanomaterials, high-throughput detection

## Abstract

As the global food industry expands and consumers demand higher food safety and quality standards, high-throughput detection technology utilizing digital intelligent optical sensors has emerged as a research hotspot in food testing due to its advantages of speed, precision, and non-destructive operation. Integrating cutting-edge achievements in optics, electronics, and computer science with machine learning algorithms, this technology efficiently processes massive datasets. This paper systematically summarizes the construction principles of intelligent optical sensors and their applications in food inspection. Sensors convert light signals into electrical signals using nanomaterials such as quantum dots, metal nanoparticles, and upconversion nanoparticles, and then employ machine learning algorithms including support vector machines, random forests, and convolutional neural networks for data analysis and model optimization. This enables efficient detection of target substances like pesticide residues, heavy metals, microorganisms, and food freshness. Furthermore, the integration of multiple detection mechanisms—including spectral analysis, fluorescence imaging, and hyperspectral imaging—has significantly broadened the sensors’ application scenarios. Looking ahead, optical sensors will evolve toward multifunctional integration, miniaturization, and intelligent operation. By leveraging cloud computing and IoT technologies, they will deliver innovative solutions for comprehensive monitoring of food quality and safety across the entire supply chain.

## 1. Introduction

Amidst the ongoing expansion and structural optimization of the global food industry, coupled with significant improvements in living standards, food quality and safety have emerged as core concerns across society. Public expectations for food safety and quality continue to rise [1]. In the field of food engineering, optical sensors have consistently served as a core technological means for ensuring food quality and safety and driving industry advancement in recent years.

Optical sensors can accurately analyze food components at the component analysis level based on the unique characteristics of various food substances, such as light absorption, scattering, and reflection. This allows for the qualitative and quantitative detection of different nutrients, additives, and other elements in food. This provides critical data support for optimizing food formulations and labeling nutritional information. For contaminant detection, whether microbial toxins, pesticide residues, or heavy metals, optical sensors can sensitively detect changes through specific optical signals, enabling timely identification of food safety risks. A 2025 study utilized perovskite heterojunctions to fabricate position sensors operating at extremely low laser powers (1–10 μW), exhibiting a sensitivity of 494.38 mV/mm to light spot displacement [2]. For freshness assessment, changes in optical properties often accompany freshness alterations during food storage and transportation. Optical sensors can monitor real-time shifts in color and gloss during fruit ripening. For instance, Alessia Cavallaro, Rossella Santonocito, and colleagues developed an optical sensor array to detect citrus fruit freshness and contamination by green mold caused by Penicillium citrinum in ripe fruits [3]. The optical array design incorporates twenty fluorescent probes capable of utilizing multiple non-covalent interactions with analytes. These probes exhibit broad absorption and emission ranges and can be excited at 365 nanometers, making them suitable for integration into citrus supply chains, including production and packaging systems. Natarajan, D and Athinarayanan, BG et al. [4] proposed a non-destructive sensor system to assess meat freshness. Meat quality is influenced by microbial populations, which correlate with carbon dioxide gas emissions and meat color. The proposed system incorporates non-dispersive infrared (NDIR), fluorescence, and color sensing technologies to capture meat CO_2_ gas and color. This provides accurate freshness information across food supply chain stages, effectively reducing food loss.

As demands for precision in food detection increase, traditional optical sensors reveal significant limitations in practical applications. Detection signals often exhibit high complexity. Since food systems are inherently complex multiphase mixtures, interactions among multiple internal components cause interference and signal superposition, making accurate extraction of target signal characteristics challenging. Fluctuations in external environmental factors such as temperature, humidity, and light intensity also readily impact detection accuracy. When confronting complex scenarios requiring simultaneous detection of multiple target substances, traditional optical sensors lack efficient signal decoupling and feature discrimination capabilities. Optical signals from different targets can easily become confused, making precise and rapid multi-target detection difficult to achieve. Traditional food testing methods, such as chemical analysis, chromatography, and mass spectrometry, offer a certain level of detection accuracy. However, they are operationally complex, time-consuming, and highly dependent on specialized personnel [5]. For large-scale food sample testing, traditional methods prove inefficient, failing to support rapid on-site detection within 30 min while also struggling to keep pace with the accelerated development of the modern food industry. Against this backdrop, digital intelligent optical sensor-based high-throughput detection technology has gained prominence in recent years for its research and application in food inspection. Integrating cutting-edge achievements from optics, electronics, and computer science, this technology enables rapid, precise, and parallel detection of multiple components and indicators in food samples [6]. With its high-throughput capability of capturing millions of molecular spectra per second, it enables rapid analysis of large sample volumes, significantly enhancing detection efficiency to meet the rapid testing demands of modern food production, distribution, and regulatory processes [7]. Additionally, this technology offers non-invasive, non-destructive testing advantages, completing analyses without compromising the original form or quality of food products—a critical benefit for high-value-added foods and those requiring integrity preservation [8].

**Figure 1:**
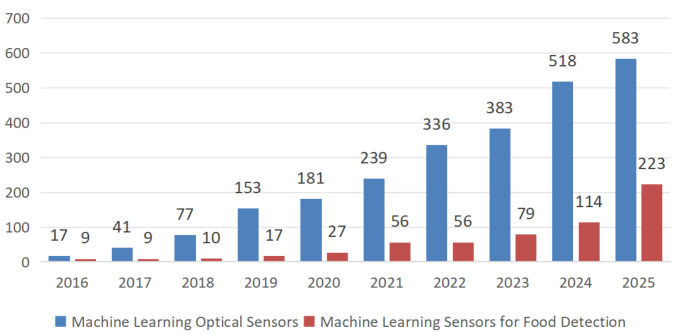
The number of publications on the application of machine learning in optical sensors for food detection in the past eight years.

Machine learning, a vital branch of artificial intelligence, plays a pivotal role in the high-throughput detection of digital optical sensors. Machine learning algorithms perform deep mining and analysis of the massive, complex data acquired by optical sensors, enabling feature extraction, pattern recognition, and classification prediction. By learning from and training on large volumes of known sample data, machine learning models can construct precise detection models, significantly enhancing detection accuracy and reliability [9]. Additionally, machine learning possesses self-optimization and adaptive capabilities. It continuously enhances detection model performance through ongoing input of new data, adapting to the evolving demands and complex, diverse environments within food inspection [10]. A keyword search in Web of Science for ‘machine learning in optical sensors’ and ‘Machine Learning Sensors for Food Detection’ reveals that, according to statistical data, the number of papers concerning the application of machine learning in optical sensors for food detection exhibited a marked upward trend between 2016 and 2024. This reflects sustained research momentum within the field. As is evident in Figure 1.

From 2016 to 2018, the development of intelligent optical sensors primarily focused on traditional machine learning approaches. Research during this period was dominated by conventional algorithms such as Random Forests (RFs), Support Vector Machines (SVMs), and Principal Component Analysis (PCA). These algorithms demonstrated strong performance in spectral classification for food detection, achieving accuracy rates exceeding 85%. The primary targets during this phase were heavy metals and pesticide residues in food. From 2019 to 2021, deep learning technologies gained prominence in optical sensors for food inspection. Convolutional neural networks (CNNs), with their robust feature extraction and image processing capabilities, played a crucial role in spectral analysis and image recognition. Image recognition accuracy using CNNs reached over 95%. This phase expanded the scope and depth of food inspection, targeting pathogenic bacteria and viruses. The study reported that CNNs were utilized to process hyperspectral images, enabling precise detection of internal defects in fruits and vegetables. Meanwhile, a 2021 study in ACS Nano successfully achieved single-molecule detection of pesticide residues by segmenting surface-enhanced Raman scattering (SERS) hotspots using U-Net, demonstrating the immense potential of deep learning in food inspection. From 2022 to 2023, the application of machine learning in optical sensors for food detection entered a new phase of integrated intelligent systems. Incorporating multimodal fusion technology, detection accuracy reached over 98%. The scope of detection targets also expanded to address emerging food safety concerns such as microplastics and antibiotic resistance.

**Figure 2:**
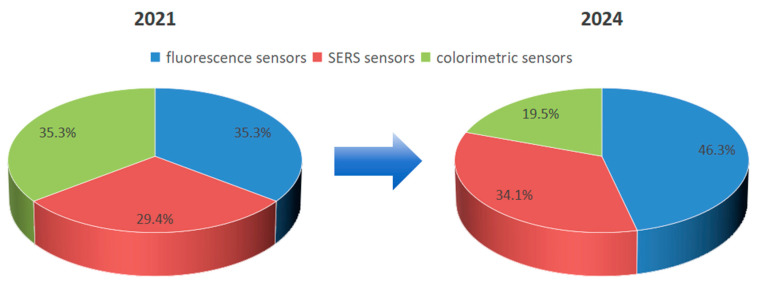
Changes in the Number of Publications Related to Three Major Categories of Optical Sensors from 2021 to 2024.

During this period, the proportion of various optical sensors used in research has also shifted significantly. This shift primarily stems from machine learning’s advantages in tackling complex spectral analysis problems, enabling broader and deeper application of fluorescence sensors in food detection. Taking the three primary detection methods as examples, a search was conducted on the Web of Science platform using the keywords: fluorescent sensors for food detection, SERS sensors for food detection, and colorimetric sensors. A pie chart was generated based on the volume of literature published in recent years, with data adjustments applied. Over the past five years, fluorescence detection has increased from 35% to 46%, surface-enhanced Raman scattering has risen from 29% to 34%, while colorimetric detection has decreased from 35% to 20%. This reflects the iterative updates and optimization of sensor technologies within the food testing sector. As is evident in Figure 2.

Machine learning-based optical sensors for food testing hold significant potential across multiple dimensions. To enhance detection performance, further tapping into the potential of machine learning algorithms to develop more efficient and precise models will significantly improve the accuracy and sensitivity of optical sensors in detecting food components and contaminants. For instance, continuously optimizing deep learning network structures enables the extraction of finer, more discriminative features from optical signals, thereby accurately identifying trace contaminants in food. Simultaneously, developing novel optical sensing materials and structures, combined with machine learning, has enabled the detection of multiple target objects within 20 min. This overcomes the challenge of multi-target detection, improves detection efficiency, and meets the high-throughput testing demands of the food industry.

Multimodal fusion represents another key development trend. Integrating optical sensors with other sensor types—such as electrochemical sensors, biosensors, and taste/smell sensors—while leveraging machine learning for comprehensive analysis of multi-source data. Different sensor types capture food information from distinct perspectives, with complementary datasets. Machine learning algorithms integrate this diverse information to construct more comprehensive and accurate models for assessing food quality and safety. For instance, optical sensors detect food appearance and optical characteristics of ingredients, electrochemical sensors measure electrical properties like conductivity, and biosensors identify specific biomarkers. Machine learning fuses these data streams to precisely judge food freshness and contamination status, providing richer and more reliable evidence for food testing.

With the rapid advancement of Internet of Things (IoT) technology, machine learning-based optical sensors will become deeply integrated into IoT ecosystems. Researchers will focus on developing compact, low-power, portable optical sensing devices while integrating machine learning algorithms to enhance device portability and miniaturization. Furthermore, to address the susceptibility of traditional optical sensor signals to environmental interference, future efforts will leverage machine learning to build more robust environmental adaptation models. Simultaneously, by analyzing correlations between vast historical environmental data and detection data through machine learning, potential patterns linking environmental factors to changes in food optical properties will be uncovered. This will provide scientific guidance for optimizing food storage and transportation conditions.

This review provides a comprehensive summary of the applications of machine learning in optical sensors, while systematically elaborating on the detection mechanisms, core components, and target analytes of optical sensors. Additionally, the future development prospects of machine learning in the field of optical sensor applications are discussed.

## 2. Development of Intelligent Optical Sensors

Traditional food safety testing methods often suffer from limitations such as complex operations, lengthy procedures, high costs, and limited sensitivity and specificity. Conventional chemical analysis requires intricate sample preparation processes and demands highly skilled technicians, while microbial detection methods necessitate extended incubation periods, making them ill suited for rapid testing requirements.

The emergence of intelligent optical sensors presents new opportunities for food safety testing. The development of intelligent optical sensors integrates machine learning methodologies, achieving significant improvements in detection accuracy and speed compared to conventional methods. During the initial sensor development phase, extensive experimental data collection captures the response characteristics of various substances to optical signals [11]. Intelligent optical sensors offer advantages such as high sensitivity, excellent selectivity, rapid response times, and user-friendly operation, enabling swift and precise detection of contaminants and harmful substances in food. Research on DNA testing for food adulteration indicates that traditional methods require several hours, whereas new automated equipment incorporating real-time fluorescent PCR technology reduces the time to under one hour. For instance, quantum dot fluorescent microspheres exhibit luminous intensities far exceeding those of conventional materials, with detection sensitivity enhanced by a factor of three to ten overall.

### 2.1. Machine Learning in Optical Sensors

Machine learning, a subfield of artificial intelligence, employs algorithms that learn patterns from data rather than executing tasks through explicit programming. These technologies learn from training datasets, identify trends across diverse databases, and perform automated data analysis [12].

Data processing is primarily achieved through the following steps. The division of the dataset is mainly accomplished by separating it into training, validation, and test sets. Dividing the data into three independent subsets forms the cornerstone of model development and evaluation. Its core purpose is to provide a completely independent, unused dataset for simulating real-world performance when adjusting the model [13]. The training set is used for learning the model’s internal parameters. The validation set facilitates hyperparameter tuning, model selection, and monitoring the training process to prevent overfitting to the training set. The test set is employed only once after all model development and tuning are complete, providing an unbiased estimate of the model’s generalization capability. Key principles for partitioning are as follows: Each subset (including training, validation, and test) should represent the overall distribution of the data. For classification problems, stratified sampling is commonly employed to ensure balanced class proportions [14].

Cross-validation enables more robust model evaluation and selection. When data volume is limited, a single fixed partition may yield unstable assessments due to randomness. The most common method is K-fold cross-validation. The training data are evenly divided into K folds. One fold is successively designated as the validation set, while the remaining K-1 folds serve as the training set, repeated K times. The final performance is averaged across the K validation results. K is typically set to 5 or 10, striking a favorable balance between computational cost and evaluation bias [15].

Core strategies to avoid overfitting: Overfitting occurs when a model excessively memorizes noise in the training data, leading to poor performance on new data. Beyond monitoring with validation sets, key techniques include the following: Regularization: Adding a penalty term to the model’s loss function to constrain parameter size, thereby reducing model complexity. Data augmentation: Applying random transformations to training data without altering labels to enhance diversity and model robustness. Ensemble methods: Constructing and combining multiple learners to perform tasks, effectively reducing variance and improving generalization ability [16].

Due to its exceptional capability in processing nonlinear data such as images, text, speech, and sensor signals, integrating machine learning can further enhance non-destructive technologies for improving food quality and safety detection [17].

Traditional machine learning refers to the application of classical algorithms and statistical techniques to analyze and interpret data, perform predictions, and automate tasks without explicitly defining programming rules [9]. It primarily learns from training data and uses this learning to predict data trends.

In food safety detection, extensive data from optical sensors must first be collected. These data may be subject to various interferences, including noise, missing values, and outliers. Therefore, data preprocessing is a critical step before analyzing and transforming the data. Machine learning algorithms can identify noise patterns within signals through learning from large datasets and perform effective noise reduction. Additionally, data standardization can further enhance stability and reliability [18].

Noise filtering, aims to eliminate random noise while preserving valid spectral features. For signals with pulsed noise or complex backgrounds, wavelet transform denoising separates noise from signal at different scales by selecting appropriate wavelet bases and thresholding strategies [19,20]. Baseline correction, employed to eliminate slow baseline drift caused by instrument background, sample scattering, or fluorescent background. Typically utilizing polynomial fitting or asymmetric least squares, baseline estimation and subtraction are performed through iterative weighted methods, proving particularly effective for spectra with complex baselines. Scattering correction and normalization: Particle size and surface roughness variations in food samples can induce severe scattering interference, causing multiplicative shifts in spectra. Standard normalized variable transformation: Each spectrum is processed individually by subtracting its mean and dividing by its standard deviation. This method effectively eliminates multiplicative effects and additive shifts caused by scattering, allowing spectra to focus more on chemical absorption features. Multivariate Scattering Correction: Assuming all spectra exhibit linear relationships with an “ideal” average spectrum, this method corrects scattering effects for each sample via linear regression. Primarily employed for diffuse reflectance spectra of solid or powdered samples. Normalization: Maximum–minimum normalization scales spectral intensity to a fixed range, suitable for pattern recognition emphasizing shape; vector normalization sets spectral magnitude to 1, similarly used to highlight shape differences [20]. Missing Data Handling: Hyperspectral images may contain missing values due to sensor defects or sample occlusion. Simple methods include filling gaps using the mean, median, or spectral dimension interpolation of neighboring pixels. More robust approaches leverage inherent data correlations, such as principal component analysis to reconstruct data in a lower-dimensional space for missing value imputation [21].

Feature extraction is a critical component of machine learning in food inspection. By analyzing preprocessed data, features reflecting food quality and safety are extracted. For optical sensor data, features such as light signal intensity, frequency, and phase can be extracted. Methods like correlation analysis and principal component analysis can then eliminate redundant or irrelevant features, improving model performance and capability [22]. Simultaneously, visual characteristics like color, shape, and texture can be leveraged using machine vision technology for feature extraction. For instance, deep learning algorithms can automatically extract effective features from food images to achieve accurate quality assessment.

Furthermore, machine learning models that have been constructed, trained, and optimized can predict and classify new food samples, enabling automatic classification and judgment of detection results [23]. For binary classification problems, they can determine whether food is qualified or contains harmful substances. For multi-class classification problems, they can categorize food by variety, grade, origin, and other attributes.

During prediction and classification, performance metrics such as accuracy, precision, recall, F1 score, ROC curve, and AUC can be used to evaluate model effectiveness. If a model’s performance fails to meet detection requirements, further parameter tuning or adoption of more complex machine learning algorithms can optimize the model to enhance accuracy and generalization capabilities [24]. Taking machine learning-based pesticide residue detection as an example, neural network models are trained on sample data to achieve high-precision, rapid detection. Simultaneously, machine learning algorithms can comprehensively evaluate food quality by analyzing optical sensor signals and chemical composition data, determining freshness and compliance with quality standards [25].

Generally, machine learning training methods and whether training data are labeled can be categorized into supervised learning and unsupervised learning [26]. Key algorithms employed in constructing intelligent optical sensors include support vector machines (SVMs), random forests (RFs), k-nearest neighbors (k-NNs), artificial neural networks (ANNs), principal component analysis (PCA), convolutional neural networks (CNNs), and autoencoders. These statistical analysis methods are integrated into the sensor’s construction and analysis processes, where they synergize with sensor components to enhance overall performance [27].

When classifying or performing regression predictions based on spectral characteristics, models such as Support Vector Machines (SVMs), Random Forests (RFs), and Artificial Neural Networks (ANNs) are widely applied.

In the same apple juice adulteration detection study, ANN also demonstrated commendable performance, though its overall capability was marginally inferior to SVM. The advantage of ANN lies in its modeling flexibility; however, its performance heavily relies on network architecture design, substantial training data, and meticulous parameter tuning, otherwise it is prone to overfitting [28].

Random forests achieve predictions by constructing multiple decision trees and integrating their outcomes. For the multivariate, interactively complex data common in optical sensing, RF typically offers a stable and readily implementable solution. However, compared to optimal SVMs or deep learning models, its predictive accuracy may occasionally plateau at a steady upper limit [29]. Machine learning techniques commonly employed in optical sensors for food inspection are shown in Table 1.

#### 2.1.1. Supervised Learning

**Figure 3:**
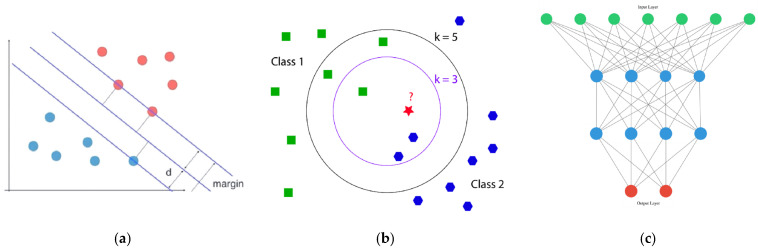
Schematic diagram of supervised learning in machine learning; (**a**) schematic diagram of the principles of support vector machines; (**b**) schematic diagram of basic principles of k-nearest neighbor; (**c**) schematic diagram of basic principles of artificial neural networks.

Building a model that maps inputs to outputs becomes the goal of the Traditional Machine Learning (TML) technique if the properties of both the input and output datasets are completely labeled. We call this guided learning [30]. For supervised learning, models can be trained using known food quality labels to learn patterns and rules within the data. This enables the establishment of mathematical models linking signals to harmful substances in food. After selecting an appropriate machine learning algorithm, models are trained using preprocessed and feature-extracted data. When new sample signals are input, the model can rapidly and accurately predict the content of harmful substances [31]. Common supervised learning algorithms include Support Vector Machines (SVMs), Decision Trees, Random Forests, and Artificial Neural Networks. In food detection, models are primarily trained using labeled data for classification and regression tasks. Key applications involve classifying food types based on spectral data and predicting food component concentrations, as is evident in Figure 3.

• Support Vector Machines (SVMs). SVM is a supervised learning algorithm used for binary classification, multi-class classification, and even outlier detection [32]. The core principle behind SVM is to find a hyperplane that maximizes the margin between data points of different classes. The data points closest to this hyperplane are termed support vectors, which play a crucial role in defining the decision boundary. For regression tasks, Support Vector Regression (SVR) is employed—a variant of SVM designed for regression problems. Its objective is to find a hyperplane that fits the data points as closely as possible while keeping the bias or error within a specified threshold. For samples that are not linearly separable, kernel functions are used to project points into a high-dimensional space to achieve linear separability [33]. SVR demonstrates a distinct advantage over SVM when predicting continuous values, finding primary application in quantitative analysis. It can significantly outperform other regression models in predictive accuracy. Cao, JQ et al. [34] present a new method using a combination of the fast Fourier transform (FFT)–support vector regression (SVR) algorithm for fast spectral demodulation of an optical fiber torsion sensor based on Sagnac interferometer (SI). Experimental results demonstrate that with the aid of the FFT-SVR algorithm, the full torsion angle range from -360 degrees to 360 degrees can be predicted with a mean absolute error (MAE) of 3.05 degrees and determination coefficient of 0.9995. Consequently, SVMs are well suited for handling nonlinear classification problems and possess the potential to process diverse types of data in array sensing detection [35]. In practical applications, training SVMs is computationally intensive, particularly when handling large datasets or complex kernel functions. Outliers can also significantly impact the placement of the decision boundary, leading to suboptimal results. Compared to other methods, SVMs offer unique advantages as they require fewer training samples to build models and are less susceptible to outliers. Consequently, it has been employed to combine harvesters with non-destructive techniques for numerous applications, such as simple and rapid classification of various foods [36], disease detection [37], and quantitative analysis of chemical components in food materials [38]. However, when applied to extremely large datasets, SVM is computationally inefficient due to the high training time required. Additionally, SVM often performs poorly when data contain noise or when categories or labels overlap.
• k-Nearest Neighbors. KNN performs classification by measuring distances between different feature values. Its core principle involves simply providing a target prediction, then calculating distances or similarities between this prediction and all samples, followed by voting on the decision using these distances or similarities. Consequently, the KNN classifier heavily relies on the K nearest values, where the parameter K significantly impacts the model’s recognition accuracy and thus requires optimization for further analysis [39]. The KNN algorithm offers advantages such as simplicity and high accuracy, making it widely applied in food variety classification, quality assessment of aquatic products and meat products, and pesticide residue detection in leafy vegetables. Furthermore, KNN serves not only as an effective classifier but also finds utility in hyperspectral imaging detection. Additionally, integrating KNN with E-nose data and machine vision images through decision layer fusion achieves higher accuracy in tea grade classification.
• Artificial Neural Networks. Artificial Neural Networks are a category of biologically inspired computational models widely applied in classification and predictive scenarios. Each individual artificial neuron functions as a basic classifier, generating a decision-related signal based on inputs received from preceding neurons in the network. Information transmission across different layers of the ANN can be facilitated through diverse transfer functions, such as sigmoid, linear, hyperbolic tangent, and logistic functions. Typically, an artificial neural network is constructed by integrating hundreds of these fundamental computational components [30]. The training process of an ANN entails inputting a dataset with predefined target outputs into the network. Learning is accomplished by reducing the discrepancy between the network’s predicted outputs and the actual desired results. Backpropagation is a common technique employed to minimize the loss function in ANN computations; It allows errors to be propagated backward through the network layers, enabling continuous adjustments to the weights and biases of the neurons [40]. Key merits of ANNs include their capability to learn and model nonlinear, complex systems, their ability to generalize patterns and relationships from limited sample data, and their flexibility with input variables; specifically, they do not impose any constraints such as assumptions regarding the distribution of input data. This enables ANN to generalize nonlinear problems and handle noise or drift more effectively than traditional statistical methods. However, ANNs require substantial datasets for training to establish a robust model. For instance, training a high-performance image classification model (such as ResNet) typically requires annotated datasets comprising millions of images (e.g., the ImageNet dataset contains approximately 1.4 million images). Moreover, these datasets must be of high quality and comprehensive in scope. For example, a model designed to detect fruit ripeness using optical sensors should be trained on spectral data or images of fruit across different varieties, growth stages, lighting conditions, and surface states (clean, dewy, or muddy). Artificial neural networks have numerous applications when integrated with detection technologies, such as combining with electronic nose systems for precise quantitative analysis of microbial contamination, using hyperspectral systems to detect food adulteration and classify peanuts, and other non-destructive techniques in food quality-related applications.
• Random Forest. Random Forest is an ensemble learning model built on the foundation of Decision Trees (DTs). As a widely used classification model, the DT features a hierarchical tree-like structure: each internal node corresponds to a specific feature, each branch denotes a decision rule or output test criterion, and each leaf node represents a categorical label [41]. In the RF framework, numerous DTs are trained independently—each utilizing a distinct subset of the training data and a unique selection of features. For classification tasks, the final prediction result is determined by majority voting among the outputs of all individual DTs. For regression tasks, it is obtained by averaging the predictions of the constituent trees. By leveraging the collective power of multiple DTs, RF effectively mitigates the overfitting issue commonly associated with a single DT, while achieving higher accuracy and stronger robustness. For example, in a study on Shigella classification, RF successfully distinguished between four different Shigella species with an 87% accuracy rate by synthesizing predictions from multiple trees and adopting a voting mechanism [42].

#### 2.1.2. Unsupervised Learning

For unsupervised learning, clustering algorithms and principal component analysis (PCA) dimension reduction algorithms can be employed. Clustering algorithms divide a dataset into distinct categories or clusters based on specific criteria, maximizing similarity among data objects within the same cluster while maximizing dissimilarity among those in different clusters [43].

The principal component analysis (PCA) dimension reduction algorithm maps high-dimensional n-dimensional features to k-dimensional features in a low-dimensional space. These k-dimensional features are entirely new orthogonal features reconstructed from the original n-dimensional features, also known as principal components. The newly generated principal components are linear combinations of the original data [44]. The PCA algorithm reduces data dimension and complexity, facilitating better data visualization.

**Figure 4:**
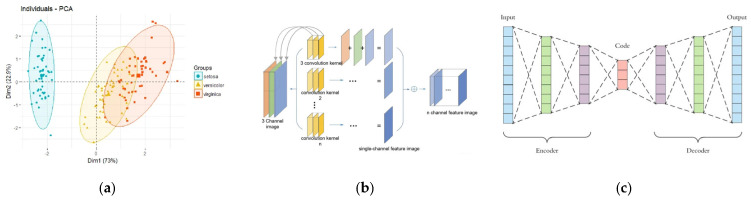
Schematic diagram of main unsupervised learning methods in machine learning; (**a**) schematic diagram of basic principles of principal component analysis; (**b**) schematic diagram of basic principles of convolutional neural networks; (**c**) schematic diagram of basic principles of automatic encoders.

In food inspection using optical sensors, PCA handles high-dimensional complex data (e.g., images, spectra), extracts deep features, and addresses nonlinear problems. It is primarily used to analyze food images, detect surface defects or foreign objects, and process high-dimensional spectral data to predict food composition or quality, as is evident in Figure 4.

• Principal Component Analysis. PCA is a widely used unsupervised algorithm in optical sensors, frequently employed to reduce the dimension of high-dimensional data by creating new variables that encapsulate the fundamental characteristics of the data in a smaller dimension [45]. In spatial analysis, each principal component captures distinct patterns representing the variation range of new variables obtained through transformations of multiple variables in the original data [46]. In practical applications, 2D or 3D PCA scatter plots based on array sensor data visualize feature relationships among similar analytes. Furthermore, PCA extracts fundamental features from samples by reducing dimensions, which greatly benefits subsequent supervised algorithms [47,48]. Additionally, as an extension of PCA, the Partial Least Squares Discriminant Analysis (PLS-DA) method utilizes predictive variables (X) and classification response variables (Y) to construct latent variables for optimal category separation [47].
• Convolutional Neural Networks. The primary machine learning algorithm applicable to signal transformation is the convolutional neural network (CNN). Digital signals after transformation may contain complex patterns, and CNNs can automatically extract features from these signals, such as waveform variations and frequency distribution patterns. By training on a large number of transformed signal samples, CNNs can learn the associations between signal patterns and food quality or composition across different food inspection scenarios [49].In recent years, convolutional neural networks (CNNs) have emerged as highly dynamic models within the machine learning domain. CNNs can autonomously learn deep features from input digital information, which can be utilized for subsequent classification or regression tasks [50]. The CNN architecture consists of multiple stages, primarily comprising three core components: convolutional layers, pooling layers, and fully connected layers [51]. Among these, convolutional layers form the core of the CNN, primarily generating arrays of features known as feature maps. This is achieved by applying two-dimensional symmetric operations to images using filter kernels, followed by nonlinear transfer functions to complete feature extraction. Pooling layers typically follow convolutional layers, primarily serving to reduce the dimensionality of feature maps. This approach effectively decreases the number of parameters within the network, thereby shortening computational time. The output processed through convolutional and pooling layers forms feature maps, which are passed as input to subsequent connection layers to complete prediction tasks [52]. The final output from the convolutional layer is converted into a one-dimensional array and connected to a fully connected layer. This layer receives the results from the convolutional process and utilizes them to classify images into different labels, functioning similarly to traditional neural networks [49]. However, CNNs also have certain limitations. Typically, CNNs require large datasets to achieve high classification accuracy. Acquiring such large-scale databases often presents significant challenges across various disciplines [51].
• Autoencoders. Autoencoders (AEs) are unsupervised neural networks trained via backpropagation. As powerful feature extraction tools, they map raw input data into feature vectors and reconstruct the original input using these vectors [53]. AEs receive raw inputs, with the encoder compressing representations that are subsequently decoded to reconstruct inputs. In deep AEs, lower hidden layers encode data while higher layers decode it, with error backpropagation driving training [30]. Due to their representational power, AEs can be stacked and layered to form deep learning networks. Several variants exist, including denoising AEs, sparse AEs, variational AEs, and contracting AEs [54]. These are invariably applied to high-dimensional data, with dimensionality reduction interpreted as PCA for a dataset. However, AEs offer greater flexibility than PCA. Furthermore, AEs permit both linear and nonlinear representations during dataset encoding, whereas only linear transformations are feasible in PCA [55]. In non-destructive testing techniques, AEs have been employed for feature extraction to enhance the predictive accuracy of calibration models. Ni et al. [56] developed a variably weighted stacked autoencoder to extract discriminative features from hyperspectral images (HSIs), which was applied to the online sorting of cotton films. In a similar vein, research has shown that when deep autoencoders are employed as a feature extraction tool in conjunction with HSIs, their performance in determining the chemical composition of dried black goji berries is comparable to, or even outperforms, that of PCA [55]. Furthermore, Huang et al. [57] proposed a computer vision system for estimating soluble solids content (SSCs) in Fuji apples at different maturity stages. This system employs stacked autoencoders to extract color features at the pixel level. Results indicate that the Stacked autoencoder (SAE)-based back propagation neural network (BPNN) model utilizing pixel-level color features achieves higher recognition rates at the feature level compared to BPNN models based solely on pure color features.

**Table 1:** Common Machine Learning Applications for Optical Sensors for Food Inspection.

ML Methods	Advantages	Attributes	Characteristic	Application Example	Ref.
Support Vector Machine	Good at handling small samples, high-dimensional data, precise classification, and strong robustness.	supervised learning	Classification and regression	Identifying honey from different origins using microscopic images of food structure	[58]
Convolutional Neural Network	Can automatically and efficiently extract image features, excelling in image detection tasks.	supervised learning	Automatic extraction of hierarchical features of data	Detecting defects on the surface of food through food appearance images.	[59]
Random Forest	Good tolerance to multi-feature data, less prone to overfitting, and has strong interpretability.	supervised learning	Classification and regression	Determine the category of food processing technology based on various optical characteristics.	[58]
K-Nearest neighbor	No training is required, does not rely on data distribution assumptions, and is applicable to a wide range of data types.	supervised learning	high computational complexity	Distinguish between different food categories by optical sensor data and determine the freshness or ripeness of food products based on optical characteristics.	[60]
Autoencoder	Strong dimensionality reduction ability, can remove noise and extract useful information.	Unsupervised learning	Nonlinear mapping, can be combined with other networks to improve performance	Extract key features from food optical sensor data for classification or detection. Identify anomalies or contamination in food products through reconstruction errors.	[61]
Principal Component Analysis	Effective dimensionality reduction, elimination of redundant information, simplifying data for easier subsequent analysis.	Unsupervised learning	Data downscaling and feature extraction	Process hyperspectral food detection data, extract key components for quality judgment.	[62]
Artificial Neural Networks	Can learn complex nonlinear relationships and adapt to various feature fusion analysis.	supervised learning	Dealing with nonlinear relationships	Determine food freshness by analyzing the appearance, spectrum, and other characteristics of the food.	[63]

### 2.2. Principles and Detection Mechanisms of Intelligent Optical Sensors

The working principle of optical sensors primarily involves sensor elements recognizing light signals, converting them into electrical signals, and outputting them in a form easily recognizable by humans. Typically, within an optical sensor, a light source emits rays toward a detection point. As light passes through the detection point, its intensity and color change. A receiver captures light signals of varying intensities after passing through the detection point and converts them into corresponding electrical signals. The electrical signals are then processed by a processor to yield measurement results. This constitutes the fundamental operating principle of optical sensors.

Common types of light sources and their wavelength ranges generally include the following: LEDs offer low cost, long lifespan, and high efficiency, though their light beams tend to diverge; laser diodes exhibit excellent monochromaticity, strong directionality, small spot sizes, and high brightness; broadband light sources possess wide spectral ranges, capable of covering ultraviolet to infrared wavelengths [64]. Wavelength ranges are categorized by light source and application as follows: ultraviolet: ozone detection, fluorescence excitation, pollutant monitoring; visible light: color recognition, ambient light sensing, imaging; near-infrared: component analysis, facial recognition, short-range LiDAR; mid-to-far infrared: gas analysis, thermal imaging, night vision [65]. Detectors convert optical signals into electrical signals, with their characteristics determining the system’s signal-to-noise ratio and dynamic range. Common detectors include photodiodes/phototransistors, offering rapid response and low cost, these are widely employed for detecting changes in light intensity; photomultiplier tubes, providing extremely high gain suitable for single-photon detection, though requiring high-voltage power supply [66].

Currently, intelligent optical sensors are primarily constructed by integrating machine learning modules into traditional sensors. This enables them to better adapt to processing large sample volumes and establish models compatible with the samples, thereby achieving faster recognition in subsequent detection. Detection efficiency is thus significantly enhanced.

#### 2.2.1. Sensors with Strong Integration with Machine Learning

Categories of optical sensors deeply integrated with machine learning. Optical sensors, leveraging diverse detection principles, have formed multiple technical branches in food inspection. The deep integration of machine learning with different types of optical sensors not only compensates for the technical shortcomings of traditional sensors but also spurs a series of efficient and precise detection solutions.

Its current application status and the state of detection equipment are primarily as follows: TOMRA Food enhances foreign object detection and quality grading in produce, potato products, seafood and similar items through optical sorters incorporating integrated AI models. This yields the following: Yield improvement: 1–2% increase; Reduced operational costs; Predictive maintenance minimizing unplanned downtime; Automated processes lowering labor expenses. ImpactVision (Shenzhen City, China) primarily employs hyperspectral imaging combined with proprietary machine learning software (in collaboration with Specim cameras) to assess fish freshness, avocado dry matter content, and detect foreign objects (sugar, flour, etc.). The manufacturer of the Specim cameras used in this study is Specim, Spectral Imaging Oy Ltd. (a Konica Minolta group company, Osaka, Japan). Detection coverage achieves 100%, replacing traditional destructive sampling (only 1–3% coverage); waste reduction contributes to a 50% decrease in food waste upstream within the supply chain.

Detection equipment capable of balancing performance and flexibility represents the primary trend in rapid testing. Research has developed a multimodal optical sensing system for automated food safety testing, integrating Raman spectroscopy and machine vision. The entire system is mounted on a 30 × 45 cm baseplate, offering considerable portability [58]. Dimensions and form factors typically range from handheld to desktop devices, varying in size from walkie-talkie dimensions to compact printers. Handheld spectrometers resemble industrial scanners or large power tools, facilitating on-site ‘point-and-shoot’ testing in warehouses, production lines, or supermarkets. They are primarily employed for quality control, safety screening, and scientific research. Rapid safety and authenticity screening: Used for on-site verification of products such as honey and olive oil, screening for pesticide residues and microbial contamination (e.g., aflatoxins) [67]. The following outlines the integration pathways and application value of machine learning with three typical optical sensors and other optical sensing technologies.

• Fluorescence Sensors. Fluorescence sensors detect substances based on the emitted fluorescence signals following excitation. Their core advantages lie in high sensitivity and multi-wavelength responsiveness, making them particularly suitable for detecting low-concentration contaminants in food. Fluorescence microscopy or imaging systems capture images of fluorescently labeled food samples. By analyzing fluorescence intensity and distribution, these techniques enable high-throughput detection and analysis of microorganisms, cells, and other targets. Fluorescence imaging utilizes microscopy or imaging systems to visualize labeled food samples. When detecting microorganisms, fluorescent dyes are typically used to label them. When illuminated by the microscope or imaging system, labeled microbes emit fluorescence. By analyzing fluorescence intensity and distribution, the quantity, location, and activity of microorganisms can be accurately determined [68]. For example, multispectral fluorescence imaging technology was employed to detect defective cherry tomatoes. Fluorescence excitation and emission matrices were used to measure defective areas, intact surfaces, and stem regions to determine optimal excitation and emission wavelengths. Two-way ANOVA analysis indicated that 410 nm was the optimal excitation wavelength for detecting defective areas. Principal component analysis (PCA) was performed on the fluorescence emission spectra of all regions at the 410 nm excitation wavelength to identify emission wavelengths for defect detection. The primary emission wavelengths used for detection were 688 nm and 506 nm. Combining fluorescence images with the determined emission bands demonstrated the feasibility of detecting defective cherry tomatoes with an accuracy exceeding 98% [69]. The integration of machine learning with fluorescence sensors primarily manifests in the deep analysis of complex fluorescence signals. In CNN analysis of fluorescence spectra for identifying foodborne pathogens, metabolites or specific markers of foodborne pathogens produce characteristic fluorescence spectra. However, in practical detection, background fluorescence from food matrices interferes with target signals, resulting in complex and overlapping spectra. Convolutional neural networks (CNNs) automatically extract subtle spectral features through multi-layer convolutional operations, effectively distinguishing target pathogens from background interference. Zielinski, B et al. achieved 97.24% classification accuracy using deep learning CNNs to classify bacterial colony images [70].
• Colorimetric Sensors. Colorimetric sensors enable qualitative or quantitative analysis by detecting color changes resulting from target substances reacting with colorimetric reagents. Their core advantages lie in visualization and portability, making them suitable for rapid on-site screening. The integration of machine learning further enhances the quantitative accuracy and scenario adaptability of colorimetric sensors. RGB Image Analysis Combined with SVM/RF for Quantitative Heavy Metal Detection: Heavy metal ions exhibit characteristic colors after reacting with specific colorimetric reagents. Capturing RGB images of the reaction system via smartphones or cameras converts color information into digital signals. Algorithms like Support Vector Machines (SVMs) or Random Forests (RFs) learn the mapping relationship between RGB values and heavy metal concentrations, eliminating subjective errors from manual visual color comparison. For example, in Cd^2+^ detection in rice, the RF model achieves quantitative errors below 3% across the 0.005–0.5 mg/kg concentration range by analyzing RGB images after color development, meeting national standard requirements. Smartphone apps integrate ML models for on-site detection: Embedding trained machine learning models into smartphone apps enables full automation of the “sampling–coloration–photography–detection” workflow. Smartphones have been integrated with rapid colorimetric sensors for heavy metal ions. Dang, KPT et al. [71] combined a bio-gold nanoparticle (AuNP) sensor with a lightbox designed for color reference and machine learning to detect Fe^3+^ ions in water. To enhance image quality, a lightbox was developed and standardized reference color values were implemented, significantly improving the performance of the machine learning algorithm. Compared to non-standardized methods (R^2^ = 0.8207), an approximate 6.7% improvement in evaluation metrics (R^2^ = 0.8207) was achieved.
• Surface-Enhanced Raman Scattering (SERS) Sensors. SERS sensors amplify target molecule Raman scattering signals via nano-enhanced substrates. Their fingerprinting characteristics and single-molecule detection capability enable precise identification of complex food components. However, signals are susceptible to substrate interference and peak overlap, making machine learning crucial for overcoming these challenges. The integration of machine learning with SERS focuses on signal interpretation and multi-target discrimination: PCA-LDA distinguishes similar toxins: SERS spectral peaks of structurally similar toxins exhibit minimal differences, making traditional methods ineffective for differentiation. Principal Component Analysis (PCA) first reduces the dimensionality of high-dimensional SERS data, retaining key features; Linear Discriminant Analysis (LDA) then classifies categories based on these reduced features. For instance, in simultaneous detection of aflatoxins B1/B2 in peanut oil, the PCA-LDA model achieved 99.2% discrimination accuracy while maintaining stable identification even at low concentrations of 0.1 μg/kg. Deep learning for deconvoluting overlapping peaks: When multiple pesticide residues coexist in food, their SERS peaks often overlap to form “mixed peaks,” leading to significant errors in traditional peak area integration methods. Deep learning models can learn the underlying structure of overlapping peaks through multi-layer nonlinear transformations, achieving peak “deconvolution.” For instance, in the simultaneous detection of five pesticides including imidacloprid and chlorpyrifos in fruits and vegetables, the DBN model achieved 96% accuracy in resolving mixed peaks, with detection limits below 0.05 mg/kg, meeting multi-residue testing requirements.
• Others. Sensors based on absorption/reflection spectroscopy (such as near-infrared and hyperspectral imaging) obtain chemical ‘fingerprint’ information by analyzing a substance’s absorption or reflection of light at specific wavelengths. This technology suite is maturely applied in non-destructive testing of internal quality and safety attributes, for instance predicting moisture, fat content and freshness in meat, and monitoring quality changes during storage of items like eggs [72]. Sensors based on elastically scattered light (e.g., Raman scattering, optical coherence tomography) obtain information about a substance’s physical structure, particle distribution, or internal defects by detecting scattered light signals resulting from elastic interactions between light and matter, without altering the wavelength. Machine learning plays a pivotal role in enhancing signal-to-noise ratios, feature extraction, and classification for such data. For instance, combining surface-enhanced Raman scattering with models like support vector machines can improve the sensitivity and specificity of detecting substances such as pesticide residues and illicit additives [73].

#### 2.2.2. Luminescence Mechanisms

Primarily categorized into three types: photoluminescence, chemiluminescence, and bioluminescence.

Photoluminescence occurs when an external light source (excitation light) illuminates a fluorophore or luminescent material. Common luminescent materials in recent years include quantum dots and dyes, where electrons in the material absorb energy, transitioning from the ground state (S_0_) to an excited state (S_1_ or higher). Subsequently, through non-radiative relaxation (vibrational relaxation), the electrons drop to the lowest vibrational energy level of the first excited state (S_1_). Finally, via radiative transition, they return to the ground state (S_0_), emitting a photon with lower energy (longer wavelength) than the excitation light (Stokes shift). Concurrently, the target analyte undergoes molecular recognition (binding, reaction, etc.), inducing microenvironmental changes such as energy transfer or electron transfer, which modulate the luminescence process (intensity, color, lifetime changes). Finally, a detector captures the emitted light signal to complete the detection.

Chemiluminescence is a process where chemical reactions provide energy to excite reaction product molecules, causing them to emit light. It does not require external excitation light sources, thus avoiding background scattering interference and achieving extremely high signal-to-noise ratios. Two chemical substances (typically substrate A and enzyme-labeled compound B) undergo a redox reaction. The energy released from this reaction is directly transferred to the reaction product molecules or specific fluorescent emitters. The excited molecules return from the excited state to the ground state, emitting photons. For example, enzyme-linked immunosorbent assays (ELISA) detect allergens, toxins (e.g., aflatoxins), and pathogens (e.g., Salmonella) in food. Antibodies targeting the analyte are often conjugated to horseradish peroxidase (HRP). Upon adding the luminol substrate, the emitted light intensity is proportional to the analyte concentration.

Bioluminescence is a specialized form of chemiluminescence, representing light phenomena generated by enzymatic reactions within living organisms. Catalyzed by luciferase, the substrate luciferin reacts with oxygen and ATP to produce excited-state oxidized luciferin, which emits light upon de-excitation. For instance, in rapid microbial detection, if a sample contains specific viable bacteria (e.g., *E. coli*), ATP produced during bacterial proliferation serves as an energy source. This ATP reacts with added luciferin/luciferase reagents to generate a light signal, enabling rapid quantitative microbial detection. This is commonly used for hygiene and quality monitoring in dairy products and beverages. For toxicity assessment, toxin-sensitive luminescent bacteria are employed; toxins inhibit bacterial metabolic activity, reducing luminescence intensity to evaluate toxicity in food or environmental samples.

### 2.3. Intelligent Detection Components

In recent years, gold and silver nanomaterials have frequently been employed in sensor construction. The optical properties of these nanomaterials change in response to environmental alterations, such as binding with target molecules, which shifts the peak position and intensity of their LSPR (Localized Surface Plasmon Resonance). These optical signal changes can be converted into measurable electrical signals or other signal formats, facilitating signal output and data processing by sensors. This process serves to transform optical signals into recognizable signals [74].

Rapid sensors employing nanomaterials as active components for food analysis offer simplicity, low cost, high specificity, and sensitivity. Such optical sensors incorporate diverse quantum dots (QDs) and carbon quantum dots (CQDs), metallic nanoparticles, upconversion nanoparticles (UCNPs), metal–organic frameworks (MOFs), etc. [75,76]. For instance, users require only a smartphone to complete testing within three minutes. The entire process necessitates neither complex laboratory equipment nor specialized operators. One method utilizing perovskite quantum dots in food safety testing achieves a cross-reactivity rate below 1%, while a sensor based on novel upconversion nanoparticles attains a detection limit of 38 nmol/L for ascorbic acid.

#### 2.3.1. Quantum Dots

Based on the wave–particle duality theory in quantum mechanics, quantum dots are nanoscale crystalline materials exhibiting properties intermediate between bulk materials and discrete atoms. For optical sensing, QDs demonstrate strong quantum confinement effects where the random motion of electrons is restricted, leading to size-, shape-, and composition-dependent optical properties. As QD size decreases, increased confinement enhances the bandgap energy, resulting in emission of higher-energy, shorter-wavelength visible light (blue light). Conversely, larger QDs possess lower bandgap energies and emit red light. Consequently, diverse QDs based on luminescence strategies are widely applied in sensing detection, bioimaging, phototherapy, and bioluminescence due to their advantages in photostability, high brightness, and tunable emission spectra [77].

However, quantum dots also present the following issues: The signal output of quantum dots may fluctuate over time or with environmental changes, primarily due to their nanoscale surface characteristics. Key factors include surface defects and ligand effects. Defective sites on the quantum dot surface act as charge carrier ‘traps’, preventing emission and thereby quenching fluorescence [78]. Many quantum dot materials, particularly high-performance perovskite quantum dots, exhibit extreme sensitivity to moisture, oxygen, heat, and light. Exposure to these environmental factors can cause irreversible degradation of their crystal structure, resulting in signal attenuation or failure [79]. Furthermore, under sustained illumination with high-energy excitation light, quantum dots may undergo photochemical reactions such as redox processes or chemical bond breaking, leading to photobleaching that compromises experimental results [80]. Furthermore, during real-sample detection, matrix effects arising from proteins, lipids, pigments, carbohydrates, and other compounds can interfere with accurate signal readout. These effects manifest as internal filtration, non-specific adsorption, or background fluorescence [81].

Typically, QDs can be categorized into metal-based QDs, carbon quantum dots (CQDs), and graphene quantum dots (GQDs) [82]. Metal-based QDs (e.g., cadmium sulfide (CdS), zinc selenide (ZnSe), and molybdenum disulfide (MoS_2_)) primarily consist of elements from Groups IV, II-VI, IV-VI, or III-V of the periodic table, exhibiting superior characteristics in high stability, strong absorption, and high quantum yield [83,84]. CQDs represent a novel class of carbon-based nanomaterials characterized by oxygen-containing surface functional groups, composed of unique quasi-spherical carbon nanoparticles and ultrafine particles (<10 nm). Due to this quantum confinement effect, CQDs exhibit tunable fluorescence, meaning their emission wavelength can be adjusted by controlling their size and surface functionalization [85]. Furthermore, their excellent photostability, high quantum yield, and biocompatibility further enhance their potential in various optoelectronic and biomedical applications [35,86]. In contrast to CQDs, GQDs (smaller than 10 nm or comprising fewer than ten graphene layers) are prepared from graphene or GO and can be readily embedded as minigraphene, exhibiting higher quantum yields. This is attributed to improved specific surface area and crystallinity, stemming from the inherent layered structure of GQDs [82]. Advancing the technological impact of quantum dots will require progress in optical sensing, encompassing quantum dot synthesis and assembly, integration with existing technological platforms, and developing effective quantum dot-specific device designs. Among these, the cost of quantum dot synthesis and assembly must be considered when preparing an optical sensor array (OSA) due to the combination of multiple sensors. As production scales up, the cost, quantity, and availability of precursors significantly impact the final cost. Taking colloidal quantum dots as an example, replacing expensive trimethylsilyl-substituted chalcogenides and atomically inefficient phosphine-based precursors with simpler substances like H_2_S and PH_3_ represents a potential cost-reduction strategy [87]. Additionally, sustainable large-scale synthesis should consider recycling the substantial organic solvents used in synthesis and quantum dot assembly to reduce costs and associated carbon footprint.

#### 2.3.2. Metal Nanoparticles

Metal nanoparticles and nanoclusters (MNPs), among the most popular nanomaterials, are prepared from common metallic elements such as Au, Ag, Pt, Cu, Pd, Re, Zn, Ru, etc. [88,89]. Due to interactions between metallic nanostructures and incident light, collective oscillations of free electrons—known as plasmonic excitations—become dominant, leading to surface plasmon resonance (SPR) [90]. When MNPs further aggregate, their free electrons resonate under external field excitation, forming local surface plasmon resonance (LSPR). This resonance phenomenon causes intense absorption of light at specific wavelengths, resulting in significant shifts in absorption spectra [91]. This LSPR property is suitable for preparing colorimetric sensor arrays due to analyte-induced aggregation or anti-aggregation interactions between the analyte and MNPs. Simultaneously, this LSPR characteristic of MNPs is susceptible to numerous factors, including MNP size, shape, composition, interparticle spacing, and the operational environment for reacting with the analyte [90,92]. Moreover, increased ionic strength causes contraction of the double electric layer on AuNP surfaces, a primary factor in MNP erosion processes. Building on this, sensor arrays based on MNPs with varying pH and ionic strength have been designed for food quality assessment and safety control [91]. Additionally, MNPs can be modified with stabilizers bearing different functional groups, inducing diverse interactions such as van der Waals forces, covalent bonds, or hydrogen bonds. Thus, when exposed to other target molecules, MNP-based colorimetric sensor arrays (CSAs) may exhibit distinct visible changes due to aggregation or characteristic surface alterations [86,88]. Moreover, ligand-stabilized MNPs bearing diverse amino, thiol, and carboxyl groups on their surfaces possess peroxidase-like activity, catalyzing reactions with chromogenic substrates such as 3,3′,5, 5′-tetramethylbenzidine (TMB), 4-chloronaphthol (4-CN), 3-amino-9-ethylcarbazole (AEC), o-phenylenediamine (OPD), 2,2′-azobis(3-ethylbenzothiazoline-6-sulfonic acid) ammonium salt (ABTS), 3,3′-diaminobenzidine (DAB). By monitoring changes in UV-vis spectra, these reactions enable quantitative and qualitative analysis [93,94]. For each type of analyte, distinct color signals can be obtained from CSA, providing a unique fingerprint for target analysis [95]. To further advance OSA, limitations of MNPs must be addressed, primarily including false-positive samples, sensitivity, portability, and reproducibility. In the future, functionalized MNPs will serve as an effective strategy for preparing highly sensitive, selective, and stable CSA. For instance, integrating aptamer technology can enhance affinity between the sensor and target, thereby improving sensitivity [96].

#### 2.3.3. Upconversion Nanoparticles (UCNPs)

UCNPs, typically composed of rare earth (RE)-doped compounds, represent a new generation of fluorophores capable of converting long-wavelength radiation into short-wavelength radiation through nonlinear optical processes [97]. The UCNP process relies on the sequential absorption of two or more photons by metastable, long-lived energy states, leading to the excitation of high-energy states that trigger UCNP emission. Due to the abundance of metastable states, ions containing f or d elements exhibit high conversion efficiency, and the resulting excited states possess the longest lifetimes. To date, the most effective upconversion phosphors reported are Yb^3+^ and Er^3+^-doped NaYF_4_ [98]. RE-doped UCNPs are typically soluble in organic solvents, making them suitable for preparation in organic phases. However, surface modification with inorganic shells or organic capping ligands is necessary for their safe use in sensor array technologies and to achieve good dispersion in water, thereby enabling tunable charge solubility and targeting diversity. These properties provide the most extensive database for indicator selection in sensor arrays [99,100]. For instance, oleic acid-capped UNCPs with four distinct emitters are hydrophobic and require multiple surface modification steps—such as protonation of oleic acid carboxyl groups, re-extraction, precipitation, and centrifugation—to facilitate their dispersion in water for subsequent use in multi-antioxidant recognition [100]. Furthermore, multicolor UNCPs modified with recognition elements (aptamers and antibodies) have been employed for various bacterial detection applications [99]. For broader utility, numerous solutions addressing poor UCNP dispersibility in water have been developed. These solutions offer advantages such as simplicity, efficiency, and safety. One versatile water solubilization method involves coating UCNPs with amphiphilic polymers, which not only enhances water solubility but also provides binding sites for target molecules. Furthermore, the luminescence intensity of UCNPs for optical sensing can be enhanced by optimizing dopant elements, designing core–shell structures, or other measures [101].

#### 2.3.4. MOFs

Metal–organic frameworks (MOFs) are highly porous crystalline materials that have garnered significant interest due to their wide-ranging applications, particularly in gas storage and separation, environmental pollution control, and biomedicine [102]. Through careful selection of ligands and metal nodes, coupled with the ability to tune pore size and shape, MOFs serve as ideal platforms for generating multidimensional luminescence [103]. To date, MOF-based luminescence detection primarily relies on mechanisms such as aggregation-induced emission (AIE), aggregation-induced quenching (ACQ), and distorted proton transfer. For an in-depth understanding of these sensing mechanisms, see [104]. To achieve effective luminescence detection, synthesizing luminescent MOFs preferably incorporates multiple light sources, e.g., metal ions, charge transfer, organic linkers, and guests [105]. Furthermore, incorporating luminescent guest materials, e.g., Ln^3+^ and dye molecules, represents a promising approach for constructing fluorinated surfactant agent (FSA) [106]. For analyte detection, the inherent porous structure and large surface area of luminescent MOFs play a crucial role in enhancing the interaction surface area for analyte enrichment. Effective target sensing occurs through luminescent MOFs when the porous structure aligns with the size and shape of the analyte. Moreover, owing to the multifunctionality provided by metal nodes and bridging joints, along with the feasibility of post-synthetic engineering and modification, MOFs and their derivatives represent one of the most suitable choices for preparing nanoenzymes [107,108]. Optimizing viable design approaches, such as selecting suitable precursors, doping heteroatoms, and introducing defects, plays a key role in enhancing the active site density and selectivity of specific catalytic reactions in MOF-engineered enzymes [107]. Moving forward, novel synthesis strategies will be introduced to prepare controllable MOFs, transcending conventional hydrothermal and solvothermal approaches to create more advanced and sustainable pathways. This will pave the way for developing structurally functional, performance-stable, high-quality MOF-based optical sensors. Furthermore, integrating molecular docking techniques enables the selection of specific metal ions and organic ligands through structural calculations and theoretical simulations to elucidate structure-property relationships. This approach facilitates the design of MOFs exhibiting remarkable stability under wet, acidic, or alkaline conditions [109].

#### 2.3.5. Other Materials

Beyond the aforementioned nanomaterials, other nanomaterials serve as sensors in food safety detection, including nanoenzymes, metal oxide nanomaterials, and organic fluorescent molecule-based nanomaterials, each offering distinct advantages. Catalytically active nanoenzymes can utilize fluorescence or visual detection, generating spectral and color differences after chemical reactions to amplify detection signals. Based on catalytic mechanisms, nanoenzymes can be primarily categorized into redox enzymes and hydrolases [110]. Metal oxide nanomaterials not only exhibit enzyme-like catalytic activity but also increase surface peroxide species due to elevated surface metal content, making them sensitive to surface chemical reactions and suitable for visual detection. Consequently, metal oxide nanomaterials are commonly employed for specific target detection through adsorption or reaction with ligands. Additionally, organic fluorescent molecular-based nanomaterials offer a combined capability of integrating the spectral tunability and biocompatibility of small-molecule organic fluorophores with the brightness, chemical, and colloidal stability of inorganic materials. Highly selective organic fluorescent molecules can be specifically designed for target detection based on different objectives. Metal oxide nanosheets, such as titanium dioxide (TiO_2_), niobium pentoxide (Nb_2_O_5_), zinc oxide (ZnO), manganese dioxide (MnO_2_), and tungsten trioxide (WO_3_), have garnered increased attention due to their large specific surface area and excellent physicochemical properties [111].

## 3. Target Substances for Detection

### 3.1. Pesticide Residues

Pesticides play an indispensable role in modern agricultural production, significantly enhancing crop yields through effective pest and disease control, thereby making crucial contributions to global food security. However, excessive and improper pesticide application has led to residue issues that pose major threats to food safety and public health [112].

Pesticide residues refer to the persistence of pesticides and their metabolites in environmental media such as agricultural products, soil, and water bodies after application. The wide variety of pesticides currently in use includes certain residues that pose significant potential hazards to human health. Organophosphorus pesticides, for example, interfere with nerve transmission by inhibiting acetylcholinesterase activity. Acute exposure can cause poisoning symptoms such as headaches, dizziness, nausea, vomiting, convulsions, and even coma; chronic exposure may lead to neurobehavioral dysfunction. Although organochlorine pesticides (such as DDT) have been banned, their persistent organic pollutant (POP) characteristics mean they persist in environmental media. Through bioaccumulation, they can enter the human body, disrupt the endocrine system, and increase cancer risks. Carbamate pesticides share a similar mechanism of action to organophosphates, while pyrethroid pesticides cause sensory abnormalities and motor disorders by affecting ion channel function in nerve cells [113].

In recent years, optical sensing technology has demonstrated significant advantages in pesticide residue detection. This technology achieves detection by analyzing optical signal changes generated from sample-light interactions, offering rapid, non-destructive, and real-time monitoring capabilities. However, optical sensing signals are susceptible to interference from multiple factors, making accurate qualitative and quantitative analysis challenging through optical methods alone.

**Figure 5:**
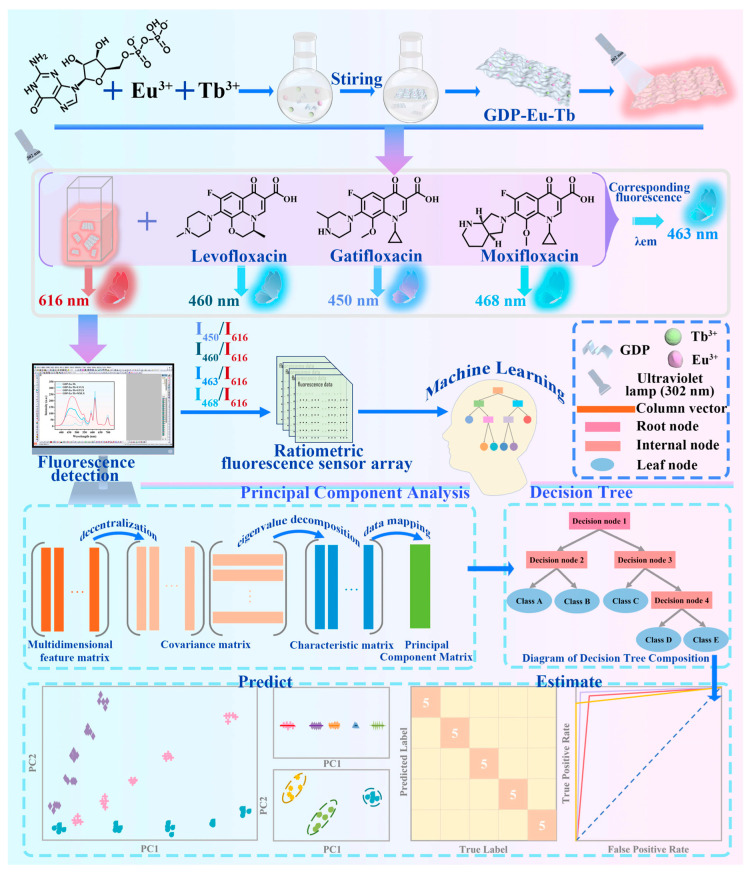
A machine learning algorithm assisted a ratiometric fluorescence sensor array based on a double-lanthanide GDP-Eu-Tb sensor in analyzing QNs [115].

**Figure 6:**
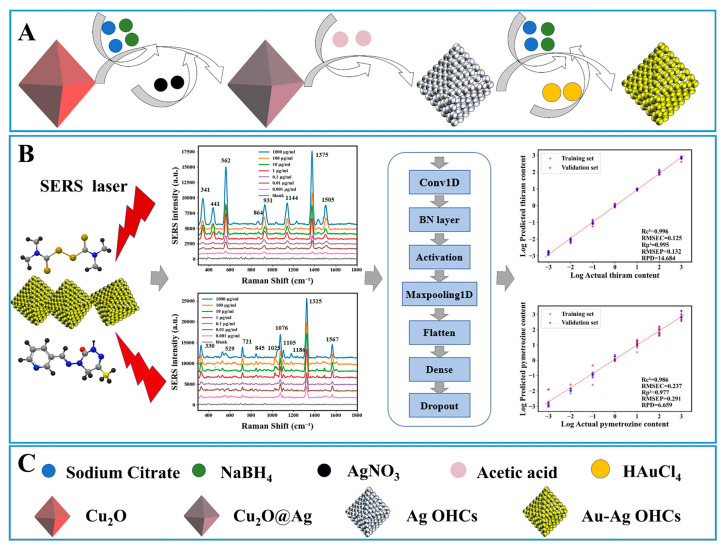
A schematic representation of the proposed method to determine thiram and pymetrozine residue in tea using SERS sensors coupled with CNNs: the preparation process of Au-Ag OHCs (**A**), data acquisition and modeling (**B**), and the material used for the preparation of the substrate (**C**) [116].

As a core technology of artificial intelligence, machine learning autonomously extracts feature patterns from massive datasets, offering innovative solutions for complex data processing and analysis. Integrating machine learning algorithms with optical sensing technology leverages their synergistic strengths: machine learning algorithms perform deep feature extraction and pattern recognition on multidimensional signals collected by optical sensors, constructing high-precision pesticide residue prediction models that significantly enhance detection accuracy and reliability. For example, A machine learning algorithm assisted a ratiometric fluorescence sensor array based on a double-lanthanide GDP-Eu-Tb sensor in analyzing QNs in Figure 5. A schematic representation of the proposed method to determine thiram and pymetrozine residue in tea using SERS sensors coupled with CNNs in Figure 6. This integrated technology offers a novel technical pathway for rapid and precise detection of pesticide residues in food, holding significant scientific research value and application potential. Therefore, conducting applied research on machine learning-based optical sensing technology for pesticide residue detection carries substantial practical significance.

High-throughput detection technology, leveraging optical sensors, enables rapid and accurate detection of pesticide residues in various food products and soil. In fruit and vegetable testing, it can precisely identify residues of multiple common pesticides, such as chlorpyrifos and cypermethrin, thereby ensuring the safety of food entering the market and preventing health risks to consumers from consuming food with excessive pesticide residues, as shown in Table 2.

### 3.2. Heavy Metals

Heavy metals are typically defined as metallic elements with a density exceeding 4.5 g/cm^3^, including lead (Pb), mercury (Hg), cadmium (Cd), chromium (Cr), and the metalloid arsenic (As). These elements are extensively used in industrial production, agricultural activities, and daily human life, inevitably entering environmental media. They accumulate within organisms through the food chain, ultimately posing serious threats to food safety [117].

The mechanisms by which heavy metals harm human health are complex and far-reaching. Lead (Pb) can cross the blood–brain barrier, disrupting normal neurotransmitter metabolism and causing irreversible damage to children’s neurological development. It can also induce anemia and renal impairment. Mercury (Hg) and its organic compounds (such as methylmercury) exhibit potent neurotoxicity, damaging the central nervous system and causing motor coordination disorders, speech impairments, and visual damage. The historically notorious Minamata disease is a classic case of methylmercury poisoning. Cadmium (Cd) primarily accumulates in the kidneys and bones within the human body. Long-term exposure can lead to renal failure, osteoporosis, and osteomalacia, and it possesses potential carcinogenicity. The toxicity of chromium (Cr) is closely related to its valence state. Hexavalent chromium (Cr(VI)), in particular, exhibits strong oxidizing properties and carcinogenicity, causing damage to the respiratory tract, skin, and digestive system. Although arsenic (As) is a metalloid, its toxicological properties resemble those of heavy metals. Long-term exposure can induce skin lesions, neurological disorders, and cardiovascular diseases, while significantly increasing the risk of malignant tumors such as skin cancer and liver cancer [118,119,120].

In recent years, optical sensing technology has demonstrated significant potential in heavy metal detection. This technology enables rapid detection by leveraging changes in absorption, scattering, or fluorescence signals resulting from interactions between heavy metal ions and optical probes. For instance, utilizing the fluorescence quenching or enhancement effects caused by the coordination of heavy metal ions with specific fluorescent reagents enables the development of highly sensitive detection methods. However, optical sensing signals are susceptible to interference from sample matrices (e.g., color, turbidity), and extracting characteristic information from complex spectra poses significant challenges, limiting its practical application in detection.

As a core technology of artificial intelligence, machine learning possesses unique advantages in complex data analysis and pattern recognition. Through autonomous learning and feature extraction, it can establish highly accurate predictive models. Integrating machine learning algorithms with optical sensing technology effectively addresses the limitations of standalone optical detection methods: machine learning algorithms can perform in-depth analysis of optical signals, eliminate matrix interference, and extract feature information correlated with heavy metal concentrations, thereby enabling high-precision quantitative analysis. This technological integration provides an innovative solution for rapid and accurate detection of heavy metals in food, holding significant scientific and practical value for enhancing food safety inspection systems and improving detection efficiency. Consequently, research on applying machine learning-based optical sensing technology to heavy metal detection represents a critical research direction in the current field of food safety, as shown in Table 3.

### 3.3. Microorganisms and Foodborne Pathogens

Microorganisms (including bacteria, fungi, viruses, etc.) are ubiquitous throughout the entire food production, processing, storage, and distribution chain. Among these, foodborne pathogenic microorganisms pose a significant threat to food safety. Foodborne pathogens refer to pathogens transmitted through the food chain that cause human disease, primarily including pathogenic bacteria, viruses, and parasites. Typical examples include enterohemorrhagic *Escherichia coli* O157:H7, *Salmonella* spp., *Listeria monocytogenes*, *Staphylococcus aureus*, and *Clostridium botulinum*. Under favorable conditions, these microorganisms can proliferate rapidly, causing food spoilage while producing toxins or directly infecting humans to trigger foodborne illnesses [130].

The mechanisms by which pathogens harm human health are complex, with severe consequences for wellbeing. Salmonella infection can cause acute gastroenteritis, progressing to sepsis in severe cases. Listeria monocytogenes exhibits strong invasiveness, penetrating the intestinal barrier, blood–brain barrier, and placental barrier to trigger meningitis, sepsis, and pregnancy-related complications. Enterotoxins produced by Staphylococcus aureus are a primary cause of food poisoning, inducing severe vomiting and diarrhea. Botulinum toxin secreted by Clostridium botulinum inhibits acetylcholine release at neuromuscular junctions, causing flaccid paralysis and potentially respiratory failure in severe cases [131].

In recent years, optical sensing technology has made significant strides in microbial detection. This approach relies on optical signal changes generated by microbial interactions with light. For instance, fluorescent labeling techniques utilize changes in fluorescent signals generated when specific recognition molecules bind to target microorganisms. Surface plasmon resonance (SPR) technology enables real-time monitoring by detecting changes in resonance angle or wavelength caused by microbial binding. However, optical sensing signals are susceptible to interference from sample matrices and environmental factors, leading to complex signal interpretation and limited detection accuracy.

Integrating machine learning algorithms with optical sensing technology fully leverages their synergistic effects: Optical sensors provide real-time detection signals, whilst machine learning algorithms utilize deep learning networks to extract features and recognize patterns from complex optical signals, effectively overcoming the limitations of traditional optical detection methods. This technological integration not only enhances detection accuracy and reliability but also significantly boosts detection efficiency, offering an innovative solution for rapid screening of microorganisms and foodborne pathogens in food. Therefore, conducting research on the application of machine learning-based optical sensing technology in microbial detection holds significant scientific and practical value for improving food safety monitoring systems and preventing outbreaks of foodborne illnesses, as shown in Table 4.

### 3.4. Other Applications

Beyond the aforementioned common targets, high-throughput optical sensor technology for food detection can also identify food additives [138], illegal additives [139], biotoxins, food freshness indicators [45,140], food allergens, and more. Food additives are widely used in the food industry; however, illegal additions and excessive usage pose serious threats to food safety. For instance, food additives are incorporated into countless commercially available foods. They enhance or impart specific flavors, extend shelf life, or achieve desired textures. It proposed an automated classification system based on the ultraviolet absorbance of five food additives. Solutions of varying concentrations were prepared by dissolving the measured additive substances in distilled water. Each substance exhibits specific absorbance peaks at particular wavelengths (e.g., acesulfame potassium shows an absorbance peak at 226 nm, while potassium sorbate correlates with a peak at 254 nm). Each additive possesses a unique spectral signature for classification [138].

Deep learning employs a multi-layered, automated feature learning mechanism, fundamentally differing from traditional machine learning which requires manually designed features.

Deep learning models (such as CNNs) can progressively abstract features from raw data through multiple convolutional and pooling layers, processing hundreds of continuous spectral and spatial bands generated by techniques like hyperspectral imaging. Deep neural networks, with their multi-layered nonlinear transformation architecture, can effectively model the complex relationship between a foodstuff’s intrinsic quality (such as sugar content, acidity, freshness) and its optical properties. For three-dimensional data like hyperspectral images, which possess both spatial and spectral dimensions, deep learning models can be designed with specific network structures to learn holistically. This approach fully exploits the integrated spatial–spectral information without requiring dimensionality reduction or splitting the data [141].

Deep learning techniques were employed for sample classification. Samples were associated with digital labels and divided into three datasets (training, validation, and testing). The CNN (Convolutional Neural Network) model achieved optimal classification results. A CNN model with three convolutional layers classified 404 spectra, yielding an average test accuracy of 92.38% ± 1.48% and an average validation accuracy of 93.43% ± 2.01% [138].

Biotoxins primarily include mycotoxins and algal toxins. During storage, food is highly susceptible to fungal contamination and toxin production. Among these, aflatoxins primarily contaminate grains, oils, and their products, exhibiting extreme toxicity and carcinogenicity; ochratoxins cause severe damage to the kidneys and liver. Different mycotoxins possess unique molecular structures, yielding specific spectral characteristics. Optical sensors collect spectral data from large numbers of food samples contaminated and uncontaminated with mycotoxins. Machine learning algorithms perform in-depth analysis and pattern recognition on this spectral data. Through model training, the algorithms can accurately extract key features associated with mycotoxins, enabling effective identification and quantitative detection of multiple mycotoxins such as aflatoxins and ochratoxins [142]. In contaminated water bodies, massive algal blooms may produce toxins. When such water sources are used for food production or aquatic organisms ingest toxin-producing algae, algal toxins enter the food chain. For instance, microcystins exhibit hepatotoxicity, posing severe threats to human health.

Food freshness indicators primarily encompass volatile compounds alongside color and texture changes. During storage and spoilage, foods release volatile substances that serve as crucial markers for freshness assessment. Taking meat as an example, trimethylamine is produced during spoilage, and its concentration correlates closely with meat freshness. An optical sensor detection system based on machine learning acquires optical signals by measuring the absorption of volatile gases emitted by meat at specific wavelengths. After collecting absorption spectral data from a large number of meat samples at varying freshness levels, machine learning algorithms analyze and model these data. The algorithm learns the complex relationship between spectral characteristics and trimethylamine content, enabling it to infer trimethylamine levels in meat and provide a scientific assessment of freshness. Fresh foods exhibit specific colors and textures; as storage time increases and spoilage occurs, their optical properties undergo significant changes. For instance, color changes in fruits can directly reflect their ripeness and freshness [143]. Optical imaging sensors capture image information of food, which contains rich features such as color and texture. CNN image recognition algorithms process and analyze this image data. By learning from a large number of images of food at different freshness levels, the algorithm can extract key features related to freshness, such as color distribution and texture complexity. Based on these features, the constructed machine learning model can accurately determine the freshness of food, providing important evidence for food quality control [144].

Food allergens are major contributors to allergic reactions in specific populations, including common examples like β-lactoglobulin (β-LG) in milk [145], ovalbumin in eggs, and certain proteins in peanuts. Surface plasmon resonance (SPR) technology based on optical sensors offers unique advantages for food allergen detection. When allergens in food bind to specific antibodies immobilized on the SPR sensor surface, they induce changes in the surface plasmon resonance signal. These signal variations are precisely measured through optical detection systems, yielding extensive SPR signal data from diverse allergen samples.

## 4. Conclusions

This study systematically recapitulates the construction principles of intelligent optical sensors and their prominent applications in food inspection, delineating a complete technical chain from signal conversion to target detection. A core technical characteristic of the proposed intelligent optical sensing system lies in the synergistic integration of nanomaterial-based signal transduction and machine learning-driven data processing: nanomaterials (e.g., quantum dots, metal nanoparticles, and upconversion nanoparticles) serve as efficient transducers to convert optical signals into detectable electrical signals, while advanced machine learning algorithms—including support vector machines, random forests, and convolutional neural networks—realize high-precision data analysis and adaptive model optimization. This integration enables the efficient and reliable detection of diverse target analytes in food systems, encompassing pesticide residues, heavy metals, pathogenic microorganisms, and indicators of food freshness, thereby providing robust technical support for food safety monitoring.

To address the increasingly complex and diverse demands of modern food inspection, the future development of intelligent optical sensors will converge toward three core directions: multifunctional integration, miniaturization, and portability. Such on-demand and real-time detection capabilities will facilitate the timely identification of potential safety hazards and the implementation of targeted corrective measures, ultimately ensuring the whole-chain safety of food from production to consumption. The in-depth integration of machine learning will further enhance the intelligence and environmental adaptability of optical sensors, marking a key development trend in this field. With the accumulation of large-scale detection data, sensors can realize continuous model optimization through online learning and transfer learning strategies; even when encountering emerging food additives or novel processing technologies, the system can quickly mine knowledge from historical data to adapt to new detection scenarios, thereby improving the generalization ability of the sensing system. Notably, the deep fusion of intelligent optical sensing systems with cloud computing and big data technologies will construct a data-driven food inspection framework with broad application prospects. This personalized service model not only enhances the practical value of detection data but also promotes the transparency and credibility of the entire food inspection system, laying a solid foundation for the establishment of a traceable and trustworthy food safety assurance system.

## Figures and Tables

**Table 2:** Optical-based on-site sensing strategies for the rapid detection of pesticide residues in agricultural foods.

Sample	Detection Method	Core Machine Learning	Effect	Testing Time	LOD	Reference
Pyrethroid pesticides (PPs) Delta, Fenva, Cyflu, Fenpro	Fluorescence	SVM, HCA	Classify PPs of different types and concentration ratios	30 min	0.047 μM, 0.06, 0.02, 0.047	[114]
QNs LVLX, GTLX, MXLX	Fluorescent	PCA, DT	Converting high-dimensional correlated data into low-dimensional, uncorrelated principal components through linear transformation for model training.	2~12 min	8.93, 9.51, 4.25 nM	[114]
Thiram, Pymetrozine	SERS	PLS, ELM, CNN	Feature extraction, data dimensionality reduction, nonlinear fitting	40~50 min	0.286 ppb 29 ppb	[114]
Fungicide: Tebuconazole, trifloxystrobin, procymidone, cymoxanil, cyazofamid	Hyperspectral Imaging (HIS)	LR, SVM, RF, CNN, PCA	Data Dimension Reduction and Preliminary Visualization Basic Classification and Feature Association High-Dimensional Feature Extraction and Optimal Classification	15–20 min	0.0125–0.0625 g/L	[114]
Imidacloprid	Fluorescence	FNN	Capturing nonlinear relationships, high-precision prediction	1.2 h	75 nM (19 μg/kg)	[114]
TC, OTC, DOX	Fluorescent	SVM	Qualitative classification, linear fitting	30–70 min	0.077, 0.075, 0.256 μM	[114]
SUs: Met, Nic, Rim, Hal, Sul	Fluorescence, colorimetry	KNN, RF, SVM, DT	Qualitative identification, integrated decision trees, anti-overfitting, kernel function mapping in high-dimensional space, recursive feature partitioning	40–60 min	0.1 μg/mL	[114]
Chlorothalonil, Carbendazim, Diazinon, Fenvalerate	Fluorescence	HCA, PCA, LDA	Clustering, Dimension Reduction, Classification Prediction	15 min	<10 ppb	[114]
TM	Smartphone-Assisted Visualization, Fluorescence	SVM, CNN	Quantitative, Visualization	55–90 min	0.1306 μmol/L (0.045 mg/kg)	[114]
Penconazole	Fluorescence	LDA, HCA			8.22 ppb	[114]

**Table 3:** Detection of heavy metals in food based on optical sensors.

Sample	Detection Method	Core Machine Learning	Effect	Testing Time	LOD	Reference
Pb^2+^	Fluorescence hyperspectral imaging	SVR (SVM Return) SDAE	Modeling and analysis results, processing deep features of data	25 min		[121,122]
Cd^2+^, Hg^2+^	Fluorescence	LDA, HCA	Qualitative discrimination, quantitative analysis; interference verification	15 min	Cd[II]: 0.501 nM; Hg[II]: 0.535 nM	[123]
Hg^2+^	Fluorescence	DL, SVM	Quantitative modeling to optimize detection accuracy	10–35 min	Fluorescence spectroscopy: 0.002 μM, smartphone: 0.834 μM	[124]
Cu^2+^, Co^2+^, Ni^2+^,Cr^3+^, Mn^2+^, Fe^3+^	Fluorescence	LDA, KNN	Dimension Reduction, Classification	30–40 min		[125]
Ni^2+^, Cr^3+^, Mn^2+^, Co^2+^, Zn^2+^, As^3+^, As^5+^, Cd^2+^, Pb^2+^	Fluorescence	RF, SVM, ANN, DT	Qualitative Classification, Quantitative Regression, Anti-Interference, Model Comparison and Selection, Image Recognition	40–45 min	5–10 μM	[126]
Cr^6+^, Fe^3+^, Fe^2+^, Hg^2+^	Fluorescence	SX-model (Stepwise Predictive Model), PCA, LDA, SVM, ANN	Prediction Logic, Unified Model Data Fusion, Model Validation and Optimization	20–25 min	1–50 μM	[85]
Fe^3+^	Colorimetry					[71]
Pb^2+^	SERS	Radial Basis Function Kernel Support Vector Machine (RBFSVM) (LR, LinSVM, NB, DT, RF, MLP) (BC, PSN, RAW) (PCA, D-tSNE)	Model Comparison, Data Preprocessing, Dimension Reduction, and Visualization		0.01–1000 μM (BACC > 80%)	[127]
Pb, Cd, Hg	Colorimetry	PLS, ELM	Regression, Model Building			[128]
Hg^2+^	Colorimetry	MLR	Build models, enhance sensitivity, reduce complexity, validate models	12 min	1 nM (0.2 ppb)	[129]

**Table 4:** Detection of foodborne bacteria based on optical techniques with the combination of different nanomaterials.

Detection Substance	Detection Method	Core Machine Learning	Effect	Testing Time	LOD	Reference
*E. coli*, *P. aeruginosa*, *S. typhimurium*, *S. aureus*, *L. monocytogenes*	Fluorescence	DT, LDA, KNN, SVM	Feature-Adaptive Selection for High-Dimensional Data Dimension Reduction Distance-Based Similarity Judgment for Nonlinear Classification	12 min	1.0 × 10^3^ CFU/mL	[132,133]
*Pseudomonas endophytica*, *Klebsiella oxytoca*, *Acinetobacter johnsonii*, *Chryseobacterium timonianum*	Colorimetry	LDA	Dimension reduction, classification, result validation, visualization analysis	6 h	102 CFU/mL	[134]
*S. aureus*, SE, *V. vulnificus*, *V. harveyi*, *L. monocytogenes*, *V. parahaemolyticus*	Colorimetry	LDA, PCA, HCA	Classification, Dimension Reduction	55 min	10 × 5 CFU/mL	[135]
Indole	Colorimetry	DCNN	Core classification model, attention mechanism modeling, residual connections to address vanishing gradients, lightweight feature extraction		3 μg/100 g	[136]
*Salmonella typhimurium*, *E. coli* O26:B6, *E. coli* O111:B4	SERS	PCA, SVC, DT, RF	Dimension reduction, model, filtering, clustering	3.5 h	μg/mL 0.7, 1.5, 1.4	[137]

## Data Availability

No new data were created or analyzed in this study. Data sharing is not applicable to this article.
